# Simultaneous multigene integration in *Aspergillus fumigatus* using CRISPR/Cas9 and endogenous counter-selectable markers

**DOI:** 10.1186/s13036-025-00539-3

**Published:** 2025-07-28

**Authors:** Luis Enrique Sastré-Velásquez, Natalia Mach, Birte Mertens, Alexander Kühbacher, Petra Merschak, Alex Dallemulle, Lukas Lechner, Clara Baldin, George Diallinas, Fabio Gsaller

**Affiliations:** 1 Institute of Molecular Biology, Biocenter, Medical University of Innsbruck, Innsbruck, Austria; 2 Research and Innovation Unit, Health University of Applied Sciences Tyrol/FH Gesundheit Tirol, Innsbruck, Austria; 3 Department of Microbiology, University of Innsbruck, Innsbruck, Austria; 4 Department of Biology, National and Kapodistrian University of Athens, Panepistimioupolis, Athens, Greece; 5 Institute of Molecular Biology and Biotechnology, Foundation for Research and Technology, Heraklion, Greece

**Keywords:** *Aspergillus fumigatus*, CRISPR/Cas9, Endogenous counter-selectable markers, Multigene integration, Azole resistance

## Abstract

**Background:**

The discovery of CRISPR/Cas9 and its subsequent accessibility in daily research initiated a new era in genome editing. This game-changing genetic instrument enabled a vast array of challenging applications requiring site-specific genome engineering as well as applications involving the equipment of cells with additional genetic traits. Despite the undisputed benefits of this technology, for facile and efficient selection of successfully manipulated cells selectable markers remain indispensable. Over the past years endogenous counter-selectable markers have come into focus in antifungal research enabling site-directed integration of multiple genes into the genome of the human mold pathogen *Aspergillus fumigatus*. However, gene cassettes had to be transformed in a consecutive manner keeping multigene integrations laborious and time-consuming.

**Results:**

In this work, we coupled the use of CRISPR/Cas9 with endogenous counter-selectable markers to achieve the simultaneous integration of multiple expression cassettes. The three markers used in this work included the herein employed *azgA* and the previously identified *fcyB* and *cntA*, responsible for 8-azaguanine, 5-fluorocytosine and 5-fluorouridine uptake, respectively. Exploiting their role in uptake of different selective agents, a triple selective transformation procedure and genomic integration of three expression cassettes in *A. fumigatus* was successfully accomplished. In addition to three distinct cellular reporters, we introduced strain-specific fluorescent reporters into four isolates displaying different levels of antifungal azole resistance to subsequently visualize and monitor their growth patterns in the same growth environment.

**Conclusions:**

The technology described in this study significantly streamlines the genetic manipulation process, reducing both time and labor associated with sequential transformations. By enabling the introduction of multiple genetic traits in a single transformation event, this strategy provides a flexible and efficient platform for a wide range of applications. As such, it enhances the potential for rapid and effective multigene integration, advancing the field of genetic engineering in fungi.

**Supplementary Information:**

The online version contains supplementary material available at 10.1186/s13036-025-00539-3.

## Introduction

Filamentous fungi are deeply integrated into the environment of animals, plants, and beyond, assuming pivotal roles in diverse habitats and making significant contributions to industrial and research endeavors [1]. Genetic manipulation tools have proven indispensable for understanding a wide array of fungal mechanisms, paving the way for novel opportunities to improve human welfare [2]. Over several decades, filamentous fungi have served as crucial industrial producers of primary and secondary metabolites. Throughout this period, genetic manipulation techniques have been employed to generate strains carrying enhanced features, aiming to maximize the biotechnological output [3]. Alternatively, manipulation of fungal genomes has significantly enhanced our understanding of target gene function, resistance mechanisms, pathobiology of fungal infections as well as virulence potential of medically relevant fungi [2].

Transformation systems commonly employed for fungal organisms have predominantly utilized auxotrophic or positive-dominant selectable markers. Auxotrophic selection requires the use of an auxotrophic recipient strain with defective biosynthetic genes, usually involved in the metabolism of nucleotides or amino acids [4, 5]. One of the major drawbacks of performing this type of selection is the necessity for a pre-existing auxotrophic mutant as parental strain which could potentially exhibit reduced virulence [6, 7]. In contrast, traditional positive-dominant selection involves the insertion of genes under a constitutive promoter, conferring resistance to growth-inhibitory compounds [4, 5, 8]. A wide range of scientists heavily rely on these genetic manipulation methods, even in light of the limited availability of drug-selectable markers and the potential for undesired phenotypes arising from the constant expression of selectable marker genes. In an effort to mitigate these shortcomings, we recently developed a new strategy using inducible positive-dominant selection in the pathogenic mold *Aspergillus fumigatus*. This involved expressing the *hph* and *ptrA* resistance genes under the xylose-inducible promoter *PxylP* [9]. In an alternative approach, negative-dominant selection, also known as counter-selection, permits the growth and selection of mutants devoid of genes involved in the uptake and/or metabolic activation of typically harmless substances such as prodrugs. The expression of a counter-selectable marker gene induces growth inhibition of the organism when cultivated in the presence of the counter-selective agent [5, 10]. Negative-dominant selection has been effectively assessed in various fungal species. Initially, genes such as those encoding thymidine kinase have been utilized as counter-selection marker, followed by selection on plates supplemented with the nucleoside analog 5-fluorodeoxyuridine [11–13]. Given the ancient loss of this enzyme in fungi [14, 15], this type of counter-selection relies on exogenous introduction of the human herpes simplex virus thymidine kinase (*hsv tk*) into the parental strain [11–13]. In recent years, the use of endogenously encoded counter-selectable markers has emerged as an appealing and innovative approach to facilitate genetic manipulations in *A. fumigatus*. This strategy involved replacement of genes responsible for uptake and key metabolic activities in the pyrimidine salvage pathway with knock-in cassettes, thereby disrupting the respective gene function and conferring resistance to different pyrimidine analogs [16, 17].

In parallel, CRISPR/Cas9-based systems stand as a powerful tool for fungal genetic engineering, having been successfully established in several filamentous fungi including *A. fumigatus* [18, 19]. Over the past few years, CRISPR/Cas9-assisted transformation in this mold has been further optimized. A system combining in vitro-assembled CRISPR/Cas9 ribonucleoproteins with microhomology repair templates has been developed in *A. fumigatus* [20]. This extends the reach of CRISPR/Cas9 technology to strains with limited Cas9 and gRNA expression, thereby reducing the likelihood of generating strains with unexpected phenotypes due to continuous Cas9 expression [21]. The remarkable efficiency of this method has been further validated, to the extent that it can even be utilized to perform selection-free genome editing, simplifying the complexity of knock-in constructions [22].

Despite these advancements, there is ample opportunity for further enhancements to revolutionize the array of molecular methodologies used in fungal genetic engineering. In this work, we assessed the potential of combining CRISPR/Cas9 technology with endogenous counter-selectable markers, aiming to facilitate and select for site-directed integration of multiple cassettes in a single transformation event. The simultaneous use of the herein described markers, each selectable with its specific agent, allowed to select for positively transformed cells harboring three knock-in constructs. Proof-of-concept of this novel multiplex approach was confirmed through integration of three cellular reporters as well as the transformation of a strain with two conditional resistance alleles together with a fluorescent reporter. The latter was employed for subsequent evaluation of azole resistance profiles of four different strains, each flagged with a different fluorescent protein, employing multicolor microscopy. Enabling targeted and rapid incorporation of numerous expression cassettes, our approach aims to advance fungal genetic engineering by promoting a wide range of research applications that require equipment of strains with multiple genetic traits.

## Materials and methods

### Growth conditions for phenotypic analyses and fungal transformation

AMM containing 20 mM ammonium tartrate as nitrogen source and 1% glucose as carbon source [23] was generally used for phenotypic analyses. Low pH AMM was prepared supplementing the medium with 0.1 M citrate buffer pH 5. Plate growth-based susceptibility assays were conducted on solid AMM comprising 1.5% agar, supplemented with either 5FC (Tokyo Chemical Industry (TCI), Tokyo, Japan), 5FU (Tokyo Chemical Industry (TCI), Tokyo, Japan), 5FUR (Tokyo Chemical Industry (TCI), Tokyo, Japan), 8AG (Carbosynth, Compton, UK) or combinations thereof. For strains carrying genes governed by *PxylP* [24], the medium was supplemented with 1% xylose to induce gene expression. Cultures were generally grown at 37 °C. Three independent experiments were carried out for phenotypical assessment.

For phenotypic analysis of strains on solid medium, 10^4^ spores of each strain were point inoculated in final volume of 5 µL of spore buffer (0.1% Tween 20, 0.9% sodium chloride solution (w/v)). Plates were generally incubated for 48 h. Solid AMM containing 1 M sucrose, 0.1 M citrate buffer pH 5 and 0.7% agar (TOP-AMM pH 5) was used to perform fungal transformations using the markers *fcyB*, *cntA* and *azgA*. Transformations using the dominant markers *hph*, *ptrA* and *ble* were carried out using TOP-AMM pH 6.5. In that case no additional buffer was supplemented to the medium. Solid Sabouraud dextrose medium (Sigma-Aldrich Corp., St. Louis, MI, USA) containing 1.5% agar was used for the generation of spores.

For fungal genetic manipulations approximately 1 µg of the respective DNAs were transformed into the corresponding recipient. Transformation and selection procedures using the dominant marker *ptrA*, *ble* and *hph* as well as counter-selectable marker *fcyB* were conducted as described previously for *A. fumigatus* [16, 25]. For *azgA-*based counter-selection, TOP-AMM pH 5 containing 10 µg/mL 8AG was used for selection. Stock solutions for 5FC (4 mg/mL) and 5FUR (20 mg/mL) were generated with water. 8AG (10 mg/mL) was dissolved in DMSO and further sonicated in a water bath (35 kHz, 1 min).

CRISPR/Cas9-assisted transformation was carried out as described previously [21] with the difference that synthetic single guide RNAs (sgRNAs; Integrated DNA Technologies, Coralville, IA, USA) containing locus-specific 20 nucleotides were used to direct Cas9 to the *fcyB* (5’-GAUUGCACCGUGUCUUAACC-3’), *cntA* (5’-GGCGGACGACAAGGCUAUGG-3’) and/or *azgA* (5’-GGCGAAACCAUCGCCCAACC-3’) locus. For design of sgRNAs the Eukaryotic Pathogen CRISPR guide RNA/DNA Design Tool [26] was used. As reference genome *Aspergillus fumigatus* A1163 (*Aspergillus fumigatus* A1163 FungiDB-58, available at FungiDB [27]) was selected to prevent any off-target hits. sgRNAs directed to the *fcyB*, *cntA* and *azgA* locus were evaluated by comparing wild type (wt; the CEA10 ∆*ku80* derivative A1160P+ [28]) protoplasts transformed with and without Cas9/sgRNA complexes (Fig S1). As repair templates, linearized reporter plasmids pESV18, pESV34 and pESV43 (Fig S2) were used targeting the *fcyB*, *cntA* and *azgA* marker locus, respectively. Employing the Cas9/sgRNA complexes yielded numerous more transformants (Fig. S1).

### Deletion of *A. fumigatus* putative *furD* orthologs and *azgA*

The strains generated and primers used in this study are listed in Table S1 and Table S2, respectively. Wt was used as parental strain. Putative *furD* orthologs AFUB_096340, AFUB_012700 and *azgA* were deleted by replacing the coding sequence of the respective gene with the *hph* cassette, conferring resistance to hygromycin B. Deletion constructs comprising approximately 1 kb of the corresponding 5´ and 3´ nontranslated regions (NTRs) linked to the central antibiotic resistance cassette were generated using fusion PCR as previously described [28].

### Generation and site-directed integration of reporter plasmids and PCR constructs at target loci

The reporter plasmids generated and used in this study are listed in Table 1. Genes encoding the green fluorescent protein variant GFP S65T [29] and the codon-adapted red fluorescent protein Katushka2S [17], under control of *PxylP*, were used as fluorescent reporters for the proof-of-concept experiment of our multigene integration strategy. In addition, a codon-optimized firefly luciferase encoding gene (*Luc*_*Opt*_) [30] was used as bioluminescent reporter. Initially, a *PxylP*-GFP(S65T) cassette was amplified from pX-sGFP [16] using the primer set pX-cass-FW/RV. This insert was fused to a backbone containing the 1-kb 5´and 3´ NTRs of *cntA*, amplified from pESV33 [17] using the primer pair BBdel-FW/RV, giving rise to pESV34. Moreover, a plasmid targeting the *azgA* locus was generated as previously described for pESV33 [17]. Briefly, the pUC19 plasmid was linearized with the primer set pUC19L-FW/RV and assembled with approximately 1-kb 5´and 3´NTRs of *azgA*, amplified from genomic DNA using the primer sets azgAN1.2-FW/azgA2.2-RV as well as azgA3.2-FW/azgAN2.2-RV, respectively, yielding pESV42. This plasmid was used as template for the BBdel-FW/RV primers to generate a specific backbone for the *PxylP* driven firefly luciferase *(Luc*_*Opt*_) gene cassette, amplified from pFG11 (Fig. S2) using the pX-cass-FW/RV primers, yielding pESV43.

**Table 1:** Reporter plasmids used for the proof-of-concept experiment

Plasmid	Used for	Reference
pESV18	Fungal transformation: ∆*fcyB*::*PxylP-Katushka2S* knock-in cassette	[17]
pESV34	Fungal transformation: ∆*cntA*::*PxylP-GFP S65T* knock-in cassette	This study
pESV43	Fungal transformation: ∆*azgA*::*PxylP-Luc*_*Opt*_ knock-in cassette	This study

Knock-in constructs containing 50 bp microhomology arms were amplified from pBM12 (*mKO2*^*PgpdA*^) [17] and pFG36 (GFP S65T^*PgpdA*^) [17] using the primer pairs 5’AfuFcyB_AnPgpdA-FW/3’AfuFcyB_AnTrpC-RV, from pFG39 (*Katushka2S*^*PgpdA*^) [17] with primer pair 5‘AfuCntA_AnPgpdA-FW/3‘AfuCntA_AnTrpC-RV, and from pΔfcyB_cyp51A^PxylP^ [31] and pΔuprt_hmg1^Tet − On(PoliC)^ [32] using the primer pairs AfuFcyB-PxylP-fw/3’AfuFcyB_AtTrpC-RV and 5’AfuAzgA_TetON(oliC)-FW/3‘AfuAzgA_AtTrpC-RV, respectively.

Reporter plasmids and PCR-generated expression cassettes containing *hmg1*^*Tet − On(PoliC)*^, *cyp51A*^*PxylP*^ and *Katushka2S*^*PgpdA*^ were transformed into wt. The *mKO2*^*PgpdA*^ (orange fluorescent protein) and *GFP S65T*^*PgpdA*^ expression cassettes were transformed into *hapE*^*P88L*^ [33] and *cyp51A*^*TR34/L98H*^ [31], respectively.

### Fluorescence and bioluminescence reporter assays

Validation of GFP S65T (excitation 473 nm; emission ≥ 510 nm) and Katushka2S (excitation 532 nm; emission ≥ 510 nm) expression of successfully transformed reporter strains was monitored with the laser scanner Typhoon FLA9500 (GE Healthcare, Chicago, IL, USA). To validate strains containing the *Luc*_*Opt*_ expression cassette, light emission was induced by supplementing 0.4 mM D-luciferin to the growth medium. The corresponding bioluminescence signal was acquired using the Fusion SL 3500 chemiluminescence imaging system (Peqlab, Erlangen, Germany).

### Multicolor fluorescence microscopy

10^4^ conidia of each *A. fumigatus* strain were combined, mixed and inoculated in 2 mL of RPMI (Sigma-Aldrich Corp., St. Louis, MI, USA) medium supplemented with 10 µg/mL doxycycline (Tokyo Chemical Industry (TCI), Tokyo, Japan), 1% xylose, with or without voriconazole (Sigma-Aldrich Corp., St. Louis, MI, USA). The conidia mixture was incubated in a µ-dish 35 mm high ibiTreat (ibidi GmbH, Gräfelfing, Germany) for 16 h at 37 °C before imaging. Voriconazole was diluted in DMSO, therefore treatment with DMSO alone was used fBor control samples.

Microscopy was performed using an inverted Olympus IX83 microscope (Olympus Austria) equipped with UCPLFLN 20XPH objective (Olympus Austria), an ibidi stage-top incubation system (ibidi GmbH, Gräfelfing, Germany), an ORCA-Flash4.0 LT Plus digital sCMOS camera (Hamamatsu Photonics, Hamamatsu, Japan), and a SpectraSplit FITC/TRITC/TexasRed/Dapi filter set (Kromnigon, Mölndal, Sweden). Three independent biological repetitions were performed for each experiment, 9 partly overlapping positions in FITC, TRITC, TexasRed and Dapi fluorescent channels were defined during live imaging without fixation of the sample. Subsequent image acquisition was performed using the semi-automatic multi-position function in cellSens Dimension software (version 3, Olympus Austria GesmbH, Vienna, Austria). Resulting 16-bit images were transferred to FiJi Software [34]. Overlapping Images were further combined into multicolor collages and pre-processed in FiJi to minimize autofluorescence and bleed-through issues using LUMoS plugin [35].

The threshold was adjusted for each channel. Fungal growth was quantified as the percentage of the image area covered by fluorescent fungal hyphae. This was determined using the standard function in Fiji: Analyze/Measure/Area Fraction, with the measurement limited to the thresholded fluorescent signal. Non-fluorescent (black) regions were considered as uncovered area. Statistical analysis was performed using two-way ANOVA and the Bonferroni tests with GraphPad Prism software 7 (GraphPad Software, San Diego, California, USA). A *p*-value ≤ 0.05 was considered statistically significant. Figures were prepared using Adobe Photoshop CC (version 19.1.7, Adobe Systems Incorporated, San José, CA, USA), graphs were prepared in GraphPad Prism software 7.

## Results

### The purine transporter encoding gene *azgA* can be used as counter-selectable marker

**Fig. 1:**
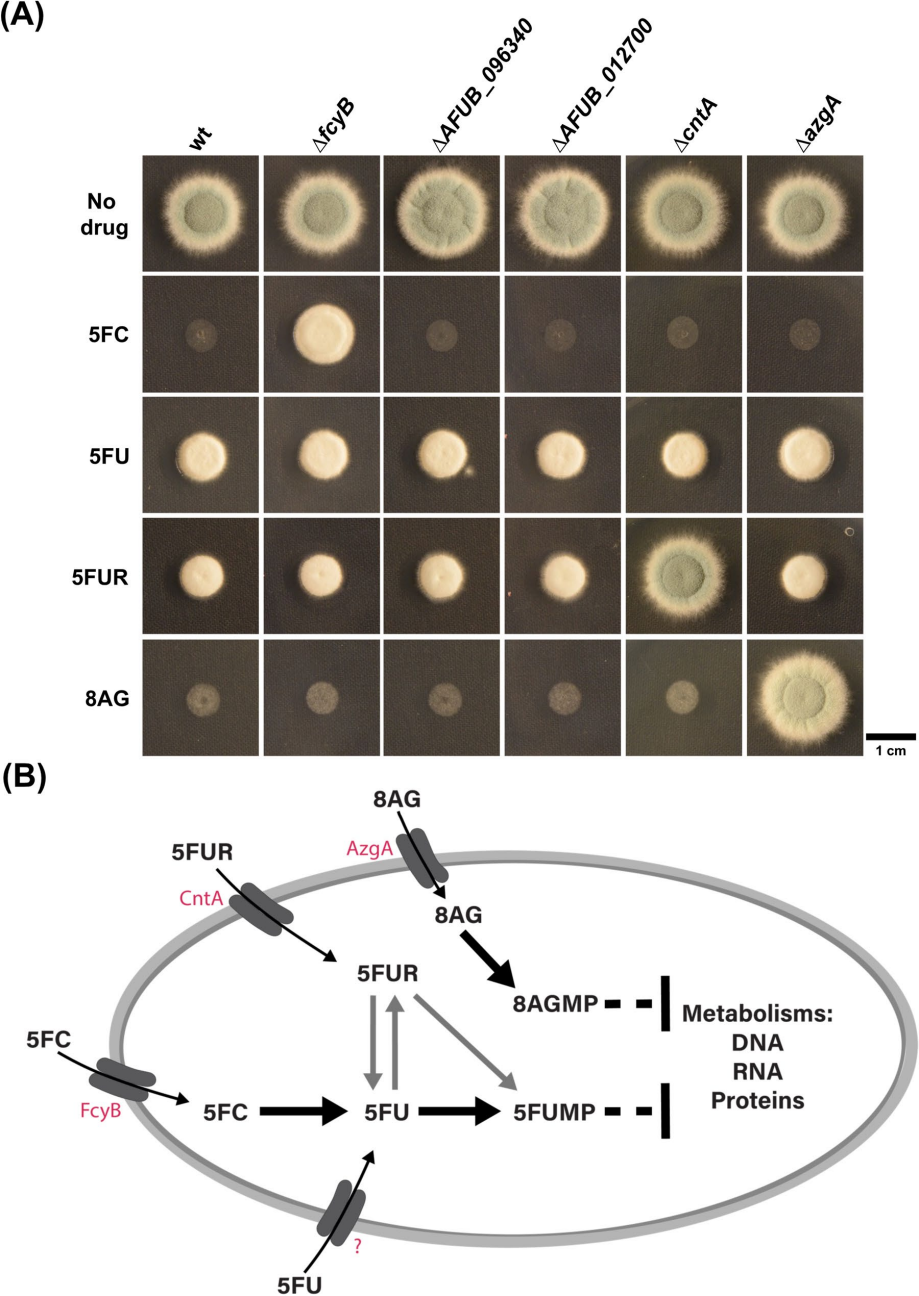
Scoping the role of different *A. fumigatus* transporters in the traffic of different nucleobase/nucleoside analogs. (**A**) Plate growth-based susceptibility testing to confirm uptake capability of strains lacking genes encoding individual transporters. Strains were point inoculated on AMM (pH 5) supplemented with 10 µg/mL of 5FC, 5FU, 5FUR or 8AG and incubated for 48 h. (**B**) Scheme illustrating the participation of *A. fumigatus* transporters in uptake and salvage of nucleobase/nucleoside analogs. The primary importer of 5-fluorouracil (5FU) in this mold remains unidentified

Several proteins mediating uptake and metabolic activation of fluorinated pyrimidines in *A. fumigatus* have been characterized in recent years [16, 17, 25]. This knowledge served as basis to develop novel counter-selection methods in *A. fumigatus*, by replacing sequences encoding FcyB, FcyA, Uprt and/or CntA with knock-in cassettes [16, 17]. To increase the possibilities of this approach, we were interested in finding additional loci to add to this genetic toolbox such as genes encoding importers of 5FU or further nucleobase/nucleoside analogous compounds. In *Aspergillus nidulans*, FurD has been described as the major importer of 5FU [36]. BLASTP analysis revealed two putative homologs of *A. nidulans* FurD in *A. fumigatus*, AFUB_096340 and AFUB_012700. In addition, disruption of the gene encoding *A. fumigatus* AzgA (AFUB_057300), encoding an adenine-guanine-hypoxanthine transporter, was shown to result in resistance to the nucleobase analog 8-azaguanine (8AG) [37]. Therefore, we speculated that *azgA* as well as *furD* orthologs AFUB_096340 and AFUB_012700 could be used as markers to perform counter-selection. To study this, we generated single knock-out mutants lacking these genes (∆*azgA*, ∆*AFUB_096340* and ∆*AFUB_012700*) in wt and monitored their growth in the presence of 5FU and 8AG. In order to rule out cross-resistance of these mutants with deletion mutants of the purine-cytosine permease encoding *fcyB* [25] and the nucleoside transporter encoding *cntA* [17], we also included the respective mutants and selecting agents 5FC and 5FUR in the assay. While mutants lacking ∆*AFUB_096340* and ∆*AFUB_012700* displayed wt-like susceptibility to all compounds, ∆*azgA* displayed clear resistance to 8AG in comparison to wt (Fig. 1A), which strongly suggested the potential use of *azgA* as a further counter-selectable marker locus for targeted insertion of knock-in cassettes. We successfully tested its use by integrating a luciferase reporter cassette at the *azgA* locus, selecting transformants in the presence of 8AG. Correct integration was validated by Southern analysis (Fig. S3).

### CRISPR/Cas9-assisted integration of three constructs into endogenous counter-selectable markers *fcyB*, *cntA* and *azgA*

In a recent study we demonstrated the successful use of four counter-selectable markers including *fcyB*, *fcyA*, *uprt* and *cntA* in a single strain [17]. However, due to overlapping roles of these marker genes in the antifungal activities of 5FC, 5FU, 5FUR, and hence resistance profiles of the corresponding mutants (Table 2), their simultaneous use for counter-selection was not feasible.

**Table 2:** Endogenous counter-selectable marker toolbox in * A. fumigatus.* Using the markers *fcyB*, *fcyA*, *uprt* and *cntA* in a consecutive order, 4 knock-in cassettes were successfully integrated at the respective loci [17]

Locus disruption	Resistance pattern	Nucleobase/Nucleoside analog used for selection (µg/mL)
***∆fcyB***	Low 5FC resistance	10
***∆fcyA***	High 5FC resistance	100
***∆uprt***	High 5FC and 5FU resistance	100
***∆cntA***	High 5FUR resistance	50*
***∆azgA***	8AG	10

*, to improve selecting conditions, 50 µg/mL clorgyline were added to the transformation medium [17]

**Fig. 2:**
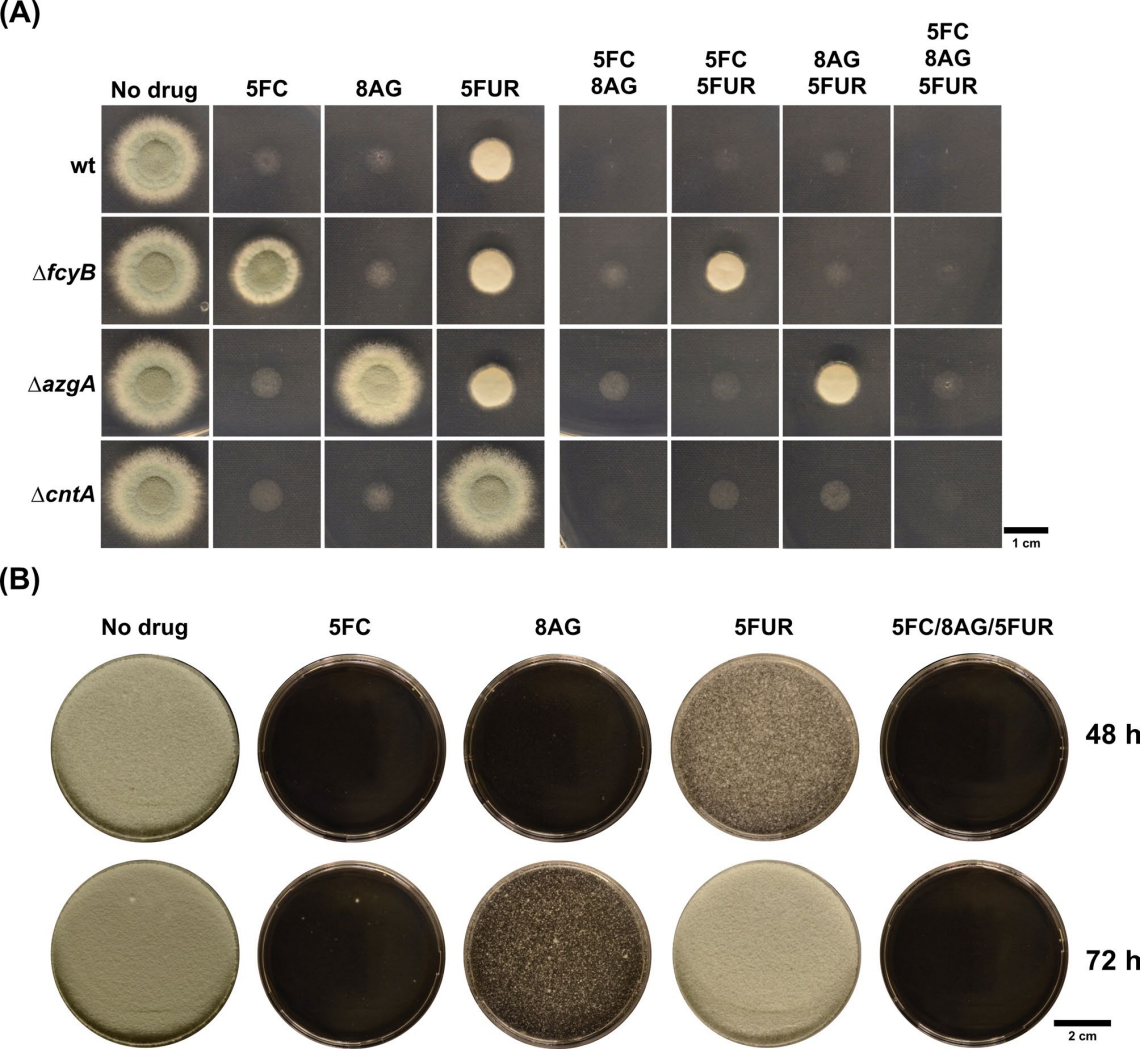
Growth of strains lacking major 5FC-, 5FUR- or 8AG-transporters can be fully abolished under combinatorial treatment. (**A**) Plate growth susceptibility testing to identify drug-specific resistance patterns of ∆*fcyB*, ∆*cntA* and ∆*azgA*. The strains were point inoculated on AMM (pH 5) and incubated for 48 h. (**B**) Growth analysis of wt protoplasts after their inoculation on TOP-AMM pH 5 supplemented with individual compounds as well as their combination. For both assays 10 µg/mL of each 5FC, 5FUR and/or 8AG was used

For instance, the disruption of *uprt* would confer resistance to high doses of 5FC as well as 5FU, therefore *fcyB* and *fcyA* had to be used prior to this marker gene to allow locus-specific selection [16]. Moreover, mutants lacking *fcyA* display 5FC hyper-resistance (> 100 µg/mL) [38], while strains with disrupted *fcyB* show slight susceptibility already at 10 µg/mL 5FC in acidic medium [16]. Therefore, the *fcyB* marker had to be employed first during sequential use. Considering that FcyB, CntA as well as AzgA mediate specific transport of 5FC, 5FUR and 8AG, respectively (Fig. 1), their simultaneous use in a multiplex approach was anticipated. To shed some light on this, two main experiments were performed. Firstly, spores of ∆*fcyB*, ∆*cntA* or ∆*azgA* mutants were point inoculated on solid AMM supplemented with 5FC, 5FUR or 8AG alone as well as with different combinations of these drugs (Fig. 2A). Secondly, wt protoplasts were generated and propagated on TOP-AMM pH 5 plates containing either 5FC, 5FUR, 8AG or all three compounds (Fig. 2B). As demonstrated previously [16, 17], at 10 µg/mL wt growth is fully blocked in the presence of 5FC but not 5FUR. 8AG led to similar growth inhibition than 5FC during the first 48 h of incubation, the time span when we expect colonies of positively transformed protoplasts to appear. Deletion mutants ∆*fcyB*, ∆*cntA* and ∆*azgA* showed a locus specific resistance pattern to 5FC, 5FUR and 8AG, respectively. Combining all three drugs fully abolished growth of point inoculated wt as well as all the other mutants on solid AMM, most likely due to the potent activity of 5FC and 8AG, or synergistic effects among all three drugs. In agreement, no growth of wt protoplasts, which we intended to use in the multiplex approach, was observed in the presence of 5FC and 8AG alone or all compounds after 48 h. This suggested that 10 µg/mL of each drug are sufficient when using *fcyB*, *cntA* and *azgA* simultaneously in a sole transformation event.

**Fig. 3:**
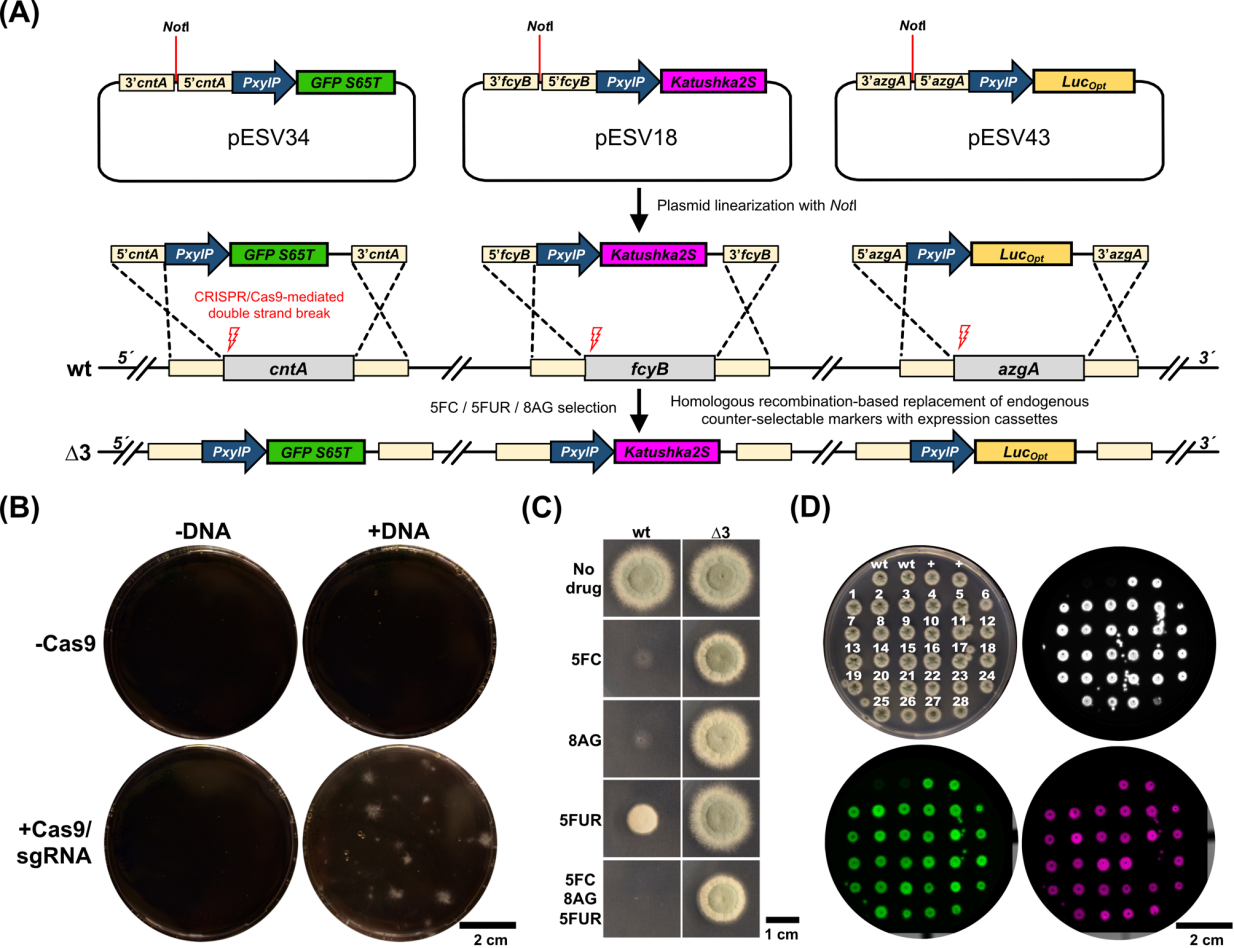
Multigene integration based on counter-selection. (**A**) Graphic illustration of the multiplex strategy followed. The plasmids pESV18 [17], pESV34 and pESV43 were *Not*I-linearized and used together in a sole transformation event, generating the multi-reporter strain *Katushka2S*^*PxylP*^*GFP S65T*^*PxylP*^*Luc*_*Opt*_^*PxylP*^ (strain ∆3). (**B**) A total of 0.5 × 10^7^ protoplasts/mL were propagated on TOP-AMM pH 5 plates containing 10 µg/mL of each 5FC, 5FUR and 8AG to select for positive transformants. Transformants only arose when wt protoplasts were transformed with all DNA templates (+ DNA) together with Cas9/sgRNAs (+ Cas9). (**C**) Plate growth susceptibility profiling of ∆3 confirmed resistance against each compound as well as their combination. The strains were point inoculated on AMM (pH 5) containing 10 µg/mL of each 5FC, 5FUR and 8AG and incubated for 48 h. (**D**) Reporter analysis of all transformants obtained from the multiplex approach to validate expression of GFP S65T (green), Katushka2S (magenta), and Luc_Opt_ (white). Spores were point inoculated on AMM pH 5 plus xylose. To visualize luciferase activity, the medium was supplemented with 0.4 mM of luciferin. +, a positive control resembling two transformants expressing all three reporters was included. The respective strain was generated during an independent transformation procedure prior to this assay

To facilitate screening of successful triple integration, *PxylP* inducible expression cassettes with distinctly detectable reporters including genes encoding GFP S65T [29], Katushka2S [17] and firefly luciferase [30] were used for integration at the individual loci (Fig. 3A). While the single use of marker loci *fcyB*, *cntA* [16, 17] and *azgA* (Fig. S1 and S3) for the integration of expression cassettes is possible with traditional protocols, their simultaneous use did not give rise to any transformants (Fig. 3B). This is most likely due to the insufficient efficiency of multigene replacements by a triple recombination event. To overcome this problem, we employed a CRISPR/Cas9 approach using locus specific sgRNAs in order to enhance the transformation efficiency. This way, we were able to generate 28 transformants (Fig. 3B). 27 transformants displayed expression of all three reporters (Fig. 3D). Repetition of the transformation and reporter assay gave rise to 26 transformants, 23 out of which were positive for the presence of all reporters (Fig S4, replicate 1). For a subset of 11 transformants correct, site-directed integration was further validated by Southern Blot analysis (Fig. S5). 5 transformants showed single copy site-specific integrations. 6 transformants displayed additional bands indicating the insertion of extra copies of the reporter cassettes. The transformation procedure was repeated in triplicate, yielding 23, 27 and 30 transformants, respectively (Fig. S4, replicates 2 to 4). Collectively, an average of 27 transformants were generated in 5 independent transformation experiments and the majority of the assayed strains showed successful expression of all three reporter genes. This demonstrates that with the aid of CRISPR/Cas9 at least three expression cassettes can be site-specifically integrated at the target loci *fcyB*, *cntA* and *azgA* in a simultaneous fashion.

Of note, by using deletion cassettes containing dominant selectable markers *ptrA*, *ble* and *hph* flanked by 5’ and 3’ homologous regions of *fcyB*, *cntA* and *azgA*, a triple knockout could be generated during selection with pyrithiamine, zeocin and hygromycin B (Fig. S6). This suggests that selecting the disruption of multiple genes can be achieved with CRISPR/Cas9 using *ptrA*, *ble* and *hph*.

### Construction of a strain with distinct conditional resistance alleles and a fluorescent reporter for simultaneous multicolor imaging

**Fig. 4:**
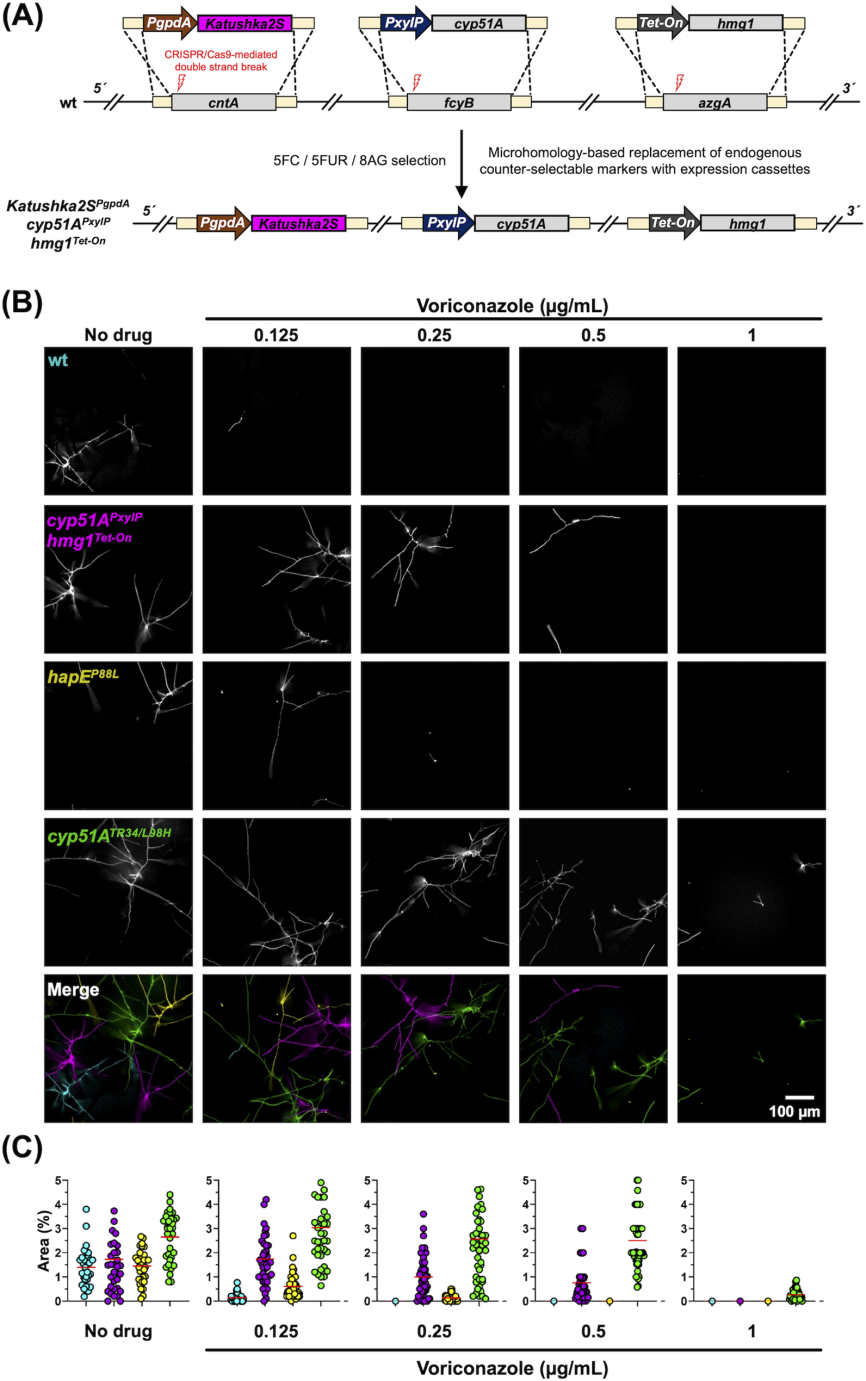
Multicolor fluorescence microscopy of 4 strains in the same growth wells. (**A**) Graphic illustration of the multigene integration strategy followed to generate *Katushka2S*^*PgpdA*^*cyp51A*^*PxylP*^*hmg1*^*Tet − On*^. (**B**) Displayed images were acquired after 16 h. Spores of *A. fumigatus* mutants carrying distinct alleles that confer azole resistance were mixed in equal concentrations and incubated for 16 h prior imaging. Strains were grown in RPMI-1640 in the presence and absence of different voriconazole concentrations. To induce *cyp51A* and *hmg1*, xylose and doxycycline were added to the medium. (**C**) Fungal growth was quantified as the percentage of area covered by individual strains

Increased expression of either *cyp51A* or *hmg1* are known to drive azole antifungal resistance in *A. fumigatus* [32, 39, 40]. To study the impact of overexpression of both genes on azole resistance, we aimed to exploit the technique established herein as well as the recently developed inducible multigene expression platform [32], to generate a strain that contains xylose- and doxycycline-inducible alleles of *cyp51A* (*fcyB* locus) and *hmg1* (*azgA* locus), respectively (Fig. 4A). Moreover, to distinguish and perform direct growth comparison of this strain in the same environment with mutants carrying the clinically relevant azole resistance-conferring mutation *hapE*^*P88L*^ (encoding a subunit of the CCAAT binding complex) [33] and the *cyp51A* resistance allele *cyp51A*^*TR34/L98H*^, we transformed wt with the two conditional resistance alleles as well as a Katushka2S gene expression cassette (*cntA* locus) [17], controlled by the constitutive promoter *PgpdA* from *A. nidulans* [41]. Pursuing a multicolor imaging-based approach, strains *hapE*^*P88L*^ and *cyp51A*^*TR34/L98H*^ were equipped with *PgpdA* driven mKO2 and GFP S65T, respectively, both at the *fcyB* locus. As azole susceptible control a wt strain harboring an mTagBFP2 expression cassette at the same locus [17], was used.

Compared to the multigene integration strategy described above, we attempted to integrate expression cassettes that were PCR-amplified from template plasmids [17]. For this, primers with tails were used that would generate repair templates containing 50 bp overlaps to the 5’ and 3’ end of the target locus, to enable CRISPR/Cas9-assisted microhomology-based recombination, similar to the previously described method by Abdallah et al. [20]. We could successfully generate the triple knock-in strain *Katushka2S*^*PgpdA*^*cyp51A*^*PxylP*^*hmg1*^*Tet − On(PoliC)*^, however, it is important to note that, compared to the linearized plasmid-based constructs comprising approximately 1 kb of 5’ and 3’ flanks of the counter-selectable markers for homologous recombination, this approach gave rise to significantly less transformants employing the same selective procedure (up to 5 in two distinct transformations). Subsequent to validation by Southern analysis (Fig. S7), microscopic analysis was carried out (Fig. 4). For this, equal spore amounts of each strain (10^4^ spores per strain) were incubated in the same well at different concentrations of voriconazole. When multicolor fluorescence microscopy was performed, strains were clearly distinguishable in merged images, allowing the measurement of hyphal growth and determination of their voriconazole-resistance patterns. The highest level of resistance was observed for *cyp51A*^*TR34/L98H*^ followed by *Katushka2S*^*PgpdA*^*cyp51A*^*PxylP*^*hmg1*^*Tet − On(PoliC)*^ when both genes were induced, and *hapE*^*P88L*^.

These results demonstrate that PCR-amplified expression modules equipped with short DNA overhangs for microhomology-mediated integration of expression cassettes using CRISPR/Cas9 can be used as knock-in cassettes to add several genetic features in a strain for downstream experimental approaches such as multicolor microscopy.

## Discussion

Despite the availability of a broad range of genetic tools that were employed for specific engineering of cells, several years ago multigene integration, particularly at specific loci, remained a daunting task. Over the past years, various endogenous counter-selectable markers were identified in *A. fumigatus* and successfully tested for targeted integrations of single genes, an entire biosynthetic cluster as well as multiple genes in single strains [16, 17, 32]. Only recently, the toolset was enlarged with *cntA*, which was exploited to integrate four genes encoding distinct fluorescent proteins, site-specifically into marker loci *fcyB*, *fcyA*, *uprt* and *cntA*, to generate a strain in which different subcellular compartments could be monitored during antifungal treatment using multicolor fluorescence microscopy [17].

Despite the apparent advantage of these markers for site-specific genomic integration of constructs, multigene integrations relied on sequential transformation events and therefore remained a laborious and time-consuming task. In this study, we aimed to overcome this hurdle by developing a technique for simultaneous integration of several cassettes in a single transformation. Although *fcyB*, *fcyA* and *uprt* worked for single as well as consecutive transformations, the disruption of each of them results in an overlapping 5FC resistance, which hampers marker-specific selection during simultaneous transformation. As a consequence, only a combination with one of these together with 5FUR selectable *cntA* would allow marker specific selection, limiting a potential combined use to two markers. The 8AG-selectable *azgA*, provided a third option for combined selection. As described above, 5FU uptake in *A. nidulans* has been mainly attributed to FurD [36], however, additional transporters contributing to the transport of uracil as well as 5FU have been revealed [42]. Disruption of either of the two putative *furD* orthologs in *A. fumigatus* did not elevate 5FU resistance (Fig. 1A). We cannot rule out that one or both of them may indeed be involved in the traffic of 5FU, but according to our data, they seem to play a negligible role in 5FU toxicity and therefore would be unsuitable as counter-selectable markers. The role of these transporters as well as the identification of a major uracil permease in *A. fumigatus* remains to be investigated.

Initial experiments testing the combined use of *fcyB*, *cntA* and *azgA* to select for equipment of cells with three DNA cassettes in the presence of 5FC/5FUR/8AG failed, most likely due to the low probability of three cassettes being site-specifically integrated by a triple recombination event in a transformant using traditional transformation protocols [17, 28]. Introducing Cas9 with locus-specific sgRNAs severely increased transformation efficiency of each locus (Fig. S1). This way, triple genomic insertions were achieved giving rise to numerous transformants (Fig. 3B and Fig. S4) with correct, site-directed integrations of gene cassettes (Fig. S5). In these first tests of the new strategy, constructs with cellular reporters flanked by approximately 1 kb of 5’ and 3’ locus specific regions for homologous recombination-mediated replacement of the endogenous markers were used. In the second test, we sought to generate a mutant using PCR-generated constructs containing only 50 bp microhomology arms. Despite a decrease in transformation efficiency, this strategy also led to triple integrations giving rise to a strain (*Katushka2S*^*PgpdA*^*cyp51A*^*PxylP*^*hmg1*^*Tet − On(PoliC)*^) with two conditional resistance alleles and a fluorescent protein tag (Fig. 4). In the demonstrated example, the respective strain was used in a multicolor fluorescence approach comparing resistance of three different strains, carrying azole resistance-associated alleles, during co-cultivation (Fig. 4).

The proof-of-concept experiments in this work illustrate only one possibility how a set of endogenous markers can be utilized for multigene integration in strains in order to stably express genes from a specific genomic site. As previously demonstrated with the introduction of a DNA cassette containing the whole penicillin biosynthetic cluster [16], consisting of approximately 17 kb of DNA, into a single locus (*fcyB*), one marker was used for the integration of several genes. Having several marker options, it is anticipated that the simultaneous integration of numerous genes, such as those of biosynthetic pathways, is feasible using the herein described technique. The strategy employed for the generation of *Katushka2S*^*PgpdA*^*cyp51A*^*PxylP*^*hmg1*^*Tet − On(PoliC)*^ highlights the modularity as a further advantage of CRISPR/Cas9 coupled counter-selection. Depending on the research requirements, a target strain can be transformed with favored combinations of expression cassettes, which are simply amplified by PCR from pre-existing DNA templates in a time- and cost-effective manner.

## Conclusions

This work outlines the development and application of a novel genetic tool, combining endogenous counter-selectable markers with Cas9/sgRNA ribonucleoprotein complexes, facilitating the site-specific integration of multiple genes into the genome. It is important to state that the endogenous markers presented here are part of the genetic makeup of *A. fumigatus* and therefore no additional, externally introduced selectable markers are needed for the integration of multiple genes. Dominant selectable markers, such as *hph*, *ble*, *ptrA*, still remain for downstream manipulations of strains. As the described endogenous selectable markers are either required to metabolize a normally harmless substance into a toxic metabolite (*fcyA*, *uprt*) or the uptake (*fcyB*, *cntA*, *azgA*) of compounds that exert growth inhibition after metabolization (Fig. 1), it is expected that orthologs as well as further genes fulfilling such or a similar requirement are present in a wide range of fungal species, but also species outside the fungal kingdom. Given the time-saving nature and modularity of the system, we anticipate that the presented method will bolster a wide range of research applications that require facile and rapid equipment of strains with multiple expression cassettes and will therefore open new avenues in fungal genetic engineering.

## Electronic supplementary material

Below is the link to the electronic supplementary material.

Supplementary Material 1

## Data Availability

No datasets were generated or analysed during the current study.
