# Supplementary material for: Simultaneous multigene integration in Aspergillus fumigatus using CRISPR/Cas9 and endogenous counter-selectable markers
Source: J Biol Eng. 2025 Jul 28;19:69. doi: 10.1186/s13036-025-00539-3 (PMC12302872; doi:10.1186/s13036-025-00539-3)
Supplement: Supplementary file 1 — Supplementary Material 1 [file 13036_2025_539_MOESM1_ESM.pdf]

Supplementary Tables and Figures

Table S1 Strains used in this study. With the exception of gene deletion mutants, all strains were generated using *in vitro* assembled Cas9-ribonucleoprotein complexes. A1160P+ illustrates the background for all mutants generated and used in this work.

Strain	Genotype	Reference
A1160P+ (wt)	Δ*ku80::pyrG*	[1]
Δ*fcyB*	Δ*fcyB::hph*	[2]
Δ*cntA*	Δ*cntA::ble*	[3]
$mTagBFP2^{PgpdA}$	Δ*fcyB::PgpdA-mTagBFP2*	[3]
Δ*AFUB_096340*	Δ*AFUB_096340::hph*	This study
Δ*AFUB_012700*	Δ*AFUB_012700::hph*	This study
Δ*azgA*	Δ*azgA::hph*	This study
Luc_{Opt}^{PxylP}	Δ*azgA::PxylP-*Luc_{Opt}	This study
$Katushka2S^{PxylP}$<br>$GFP\ S65T^{PxylP}$<br>Luc_{Opt}^{PxylP}	Δ*fcyB::PxylP-Katushka2S*<br>Δ*cntA::PxylP-GFP S65T*<br>Δ*azgA::PxylP-*Luc_{Opt}	This study
$cyp51A^{PxylP}$<br>$hmg1^{Tet\text{-}On(PoliC)}$<br>$Katushka2S^{PgpdA}$	Δ*fcyB::PxylP-cyp51A*<br>Δ*azgA::Tet-On(PoliC)-hmg1*<br>Δ*cntA::PgpdA-Katushka2S*	This study
$cyp51A^{TR34/L98H}$<br>$GFP\ S65T^{PgpdA}$	Δ*cyp51A::ble, Pcyp51A*^{TR34}*-cyp51A*^{L98H}*, hph,*<br>Δ*fcyB::PgpdA-GFP S65T*	This study
$hapE^{P88L}$<br>$mKO2^{PgpdA}$	Δ*hapE::hph-hapE*^{P88L}<br>Δ*fcyB::PgpdA-mKO2*	This study
Δ*fcyB*Δ*cntA*Δ*azgA*	Δ*fcyB::ptrA*<br>Δ*cntA::ble*<br>Δ*azgA::hph*	This study

Table S2 Primers used in this study. DNA overhangs for microhomology-based recombination are highlighted in red.

Primer set	Sequence (5´ → 3´)
hph-FW	CCGGCTCGGTAACAGAACTAACGGCGTAACCAAAAGTCAC
hph-RV	GGGAGCATATCGTTCAGAGCTCTTGACGACCGTTGATCTG
AFUB_096340-1	TCCTGGTCTCTATGCCGTTC
AFUB_096340-2	TAGTTCTGTTACCGAGCCGGGCGCACTCCAGTAACACAAC
AFUB_096340-3	GCTCTGAACGATATGCTCCCTATTCCGCGTGCGATCTAGT
AFUB_096340-4	GCTGGACTGACCACCTTCTT
AFUB_096340-N1	GGCTGAGTTGGCATTTCATT
AFUB_096340-N2	TCGCGACCAAAGTCATAGAA
AFUB_012700-1	GTCACGCTGATTGGGTACG
AFUB_012700-2	TAGTTCTGTTACCGAGCCGGTATCAAGGGAGGGATGATGC
AFUB_012700-3	GCTCTGAACGATATGCTCCCAACGCGTGTCCTATCTGGAG
AFUB_012700-4	ATTCACCCACCACGTCTCTC
AFUB_012700-N1	ACTGGGCGAAGATGGTAGTG
AFUB_012700-N2	TCCTCCCCCTGATCCTAGAC
azgA-1	TATGAACTGTAGCGGCACGA
azgA-2	TAGTTCTGTTACCGAGCCGG CAGCCTGAAGCCAGACAGTT
azgA-3	GCTCTGAACGATATGCTCCC CACCACGCCTAGGTAATTCG
azgA-4	AGAGTTGGATTTCGGACAGG
azgA-N1	GGAGATGATAGGGGCGATTT
azgA-N2	TCTTCGAGCTGGTTGACCTT
fcyB-1	CGCTATCCCAGCAATAGAGC
fcyB-2r	TAGTTCTGTTACCGAGCCGGACTGAGTCAATCCCCACCAC
fcyB-3	GCTCTGAACGATATGCTCCCTGCGGTTTTTGGGTTTTATC
fcyB-4r	CACACTGGGTCTGAAGACGA
fcyB-N1	CAGAGAATTGCCAAGCTGGT
fcyB-N2	GCGGTATGAAACAACGGTCT
cntA-1	ACTGGGGCTTTTTCTGGACT
cntA-2	TAGTTCTGTTACCGAGCCGGTTAAGAACGCGACGACCTTT
cntA-3	GCTCTGAACGATATGCTCCCTGCCTGCAAATCACAAGAAC
cntA-4	ATACATCGTCCACGGAGAGC
cntA-N1	TTTAACGCGACGACAGAATG
cntA-N2	CAAGGTGGGTGGATTTGTCT
BBdel-FW	TGTGAAATTGTTATCCGCTCACAA
BBdel-RV	AAACAGCTATGACCATGATTACGC
pX-cass-FW	AATCATGGTCATAGCTGTTTCTGATGCGAGCAACAGTATGC
pX-cass-RV	GAGCGGATAACAATTTCACATGAGGGTTGAGTACGAGATTGG
pX-FW.2	CCATGGCAGCAGTGATTTCA

pX-RV.2	GGTTGGTTCTTCGAGTCGATG
pX-K2S-FW	ATCGACTCGAAGAACCAACCATGGTCGGCGAGGACTCC
pX-K2S-RV	TGAAATCACTGCTGCCATGGCTAGGAGTGGCCCAGCTTG
pUC19L-FW	GGCATGCAAGCTTGGCGTAA
pUC19L-RV	GTACCGAGCTCGAATTCACTG
azgAN1.2-FW	CTCTGCGGCCGCGTTTAAACTATGAACTGTAGCGGCACGA
azgA2.2-RV	TTACGCCAAGCTTGCATGCC CAGCCTGAAGCCAGACAGTT
azgA3.2-FW	AGTGAATTCGAGCTCGGTAC CACCACGCCTAGGTAATTCG
azgAN2.2-RV	CATAGTTTAAACGCGGCCGC AGAGTTGGATTTCGGACAGG
5‘AfuCntA_AnPgpdA-FW	TTCTTCAAAGAAGGCGTTTTGGACACCCTGAAAGGTCGTCGCGTTC TTAAGCCGTCGGCGAAATAGCA
3‘AfuCntA_AnTrpC-RV	AATTCGCAAAATGAATATGATAAAAATACAGTTCTTGTGATTTGCAG GCATCTTGACGACCGTTGATCTG
AfuFcyB-PxylP-fw	TATACTCTTATCATTAACTTCATTTCTCCTCCCCAGGTGGTGGGGATT GACTGATGCGAGCAACAGTATGC
3‘AfuFcyB_AtTrpC-RV	CGACAAACAAGACCAATTGATAAAACCCAAAAACCGCACACTTCTA TCATAGGGTTGAGTACGAGATTGGGG
5’AfuAzgA_TetON(oliC)-FW	ATCTGTGTACGGGTTTTTCCCTCCGTGACTAACTGTCTGGCTTCAGG CTGTCCCGTAATCAATTGGCTTCGA
3‘AfuAzgA_AtTrpC-RV	ATATGGCATCCGAGTCATCAGCTCGAGATGCGAATTACCTAGGCGTG GTGAGGGTTGAGTACGAGATTGGGG
5'AfuFcyB_AnPgpdA-FW	TATACTCTTATCATTAACTTCATTTCTCCTCCCCAGGTGGTGGGGATT GAgccgtcggcgaaatagca
3'AfuFcyB_AnTrpC-RV	CGACAAACAAGACCAATTGATAAAACCCAAAAACCGCACACTTCTA TCATtcttgacgaccgttgatctg

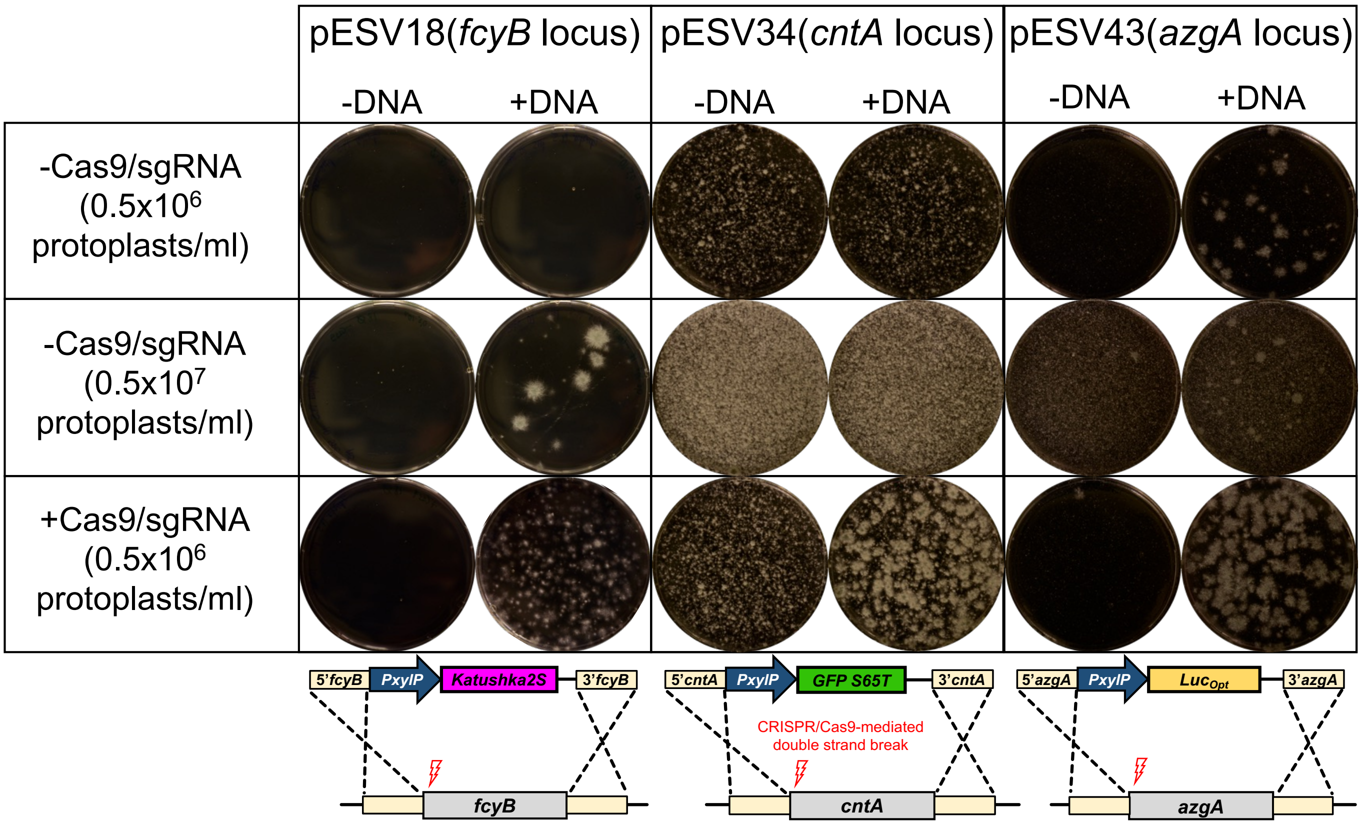

Fig S1 Comparison of fungal transformations without and with the use of Cas9/sgRNA complexes. wt was transformed with linearized reporter plasmids (see also Fig S2) targeting the *fcyB*, *cntA* and the *azgA* locus. The number of transformants increased drastically with the aid of Cas9/sgRNA complexes. Protoplasts were propagated on TOP-AMM plates containing either 10 µg/mL 5FC (*fcyB* locus), 50 µg/mL 5FUR + 50 µg/mL clorglyine (*cntA* locus) and 10 µg/mL 8AG (*azgA* locus) to select for positive transformants.

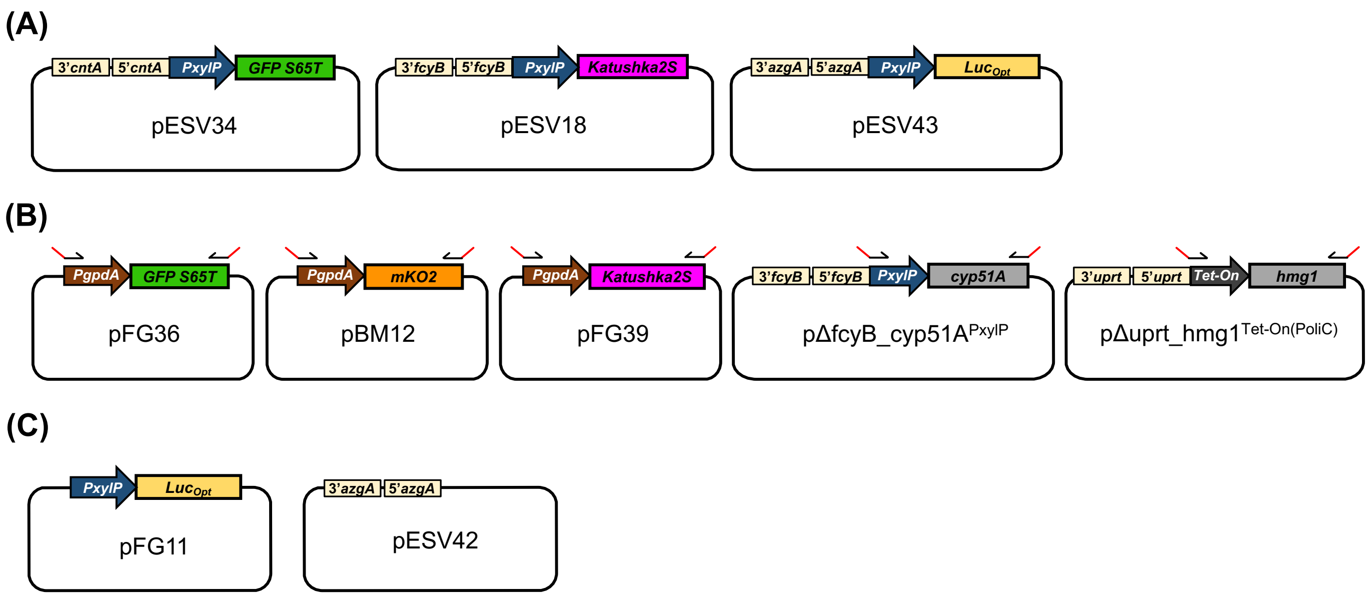

Fig S2 Schemes of main plasmids used in this work. Reporter plasmids (A) were *Not*I-linearized and directly used for transformation. Plasmids used as PCR templates (B) to generate repair templates with 50 bp overhangs (red tails) targeting 5' and 3' regions of a specific target locus for CRISPR/Cas9-assisted microhomology-based recombination. (C) pFG11 (unpublished) was used as template to amplify the *PxylP*-inducible Luc_{Opt} cassette for assembly into pESV42, yielding pESV43.

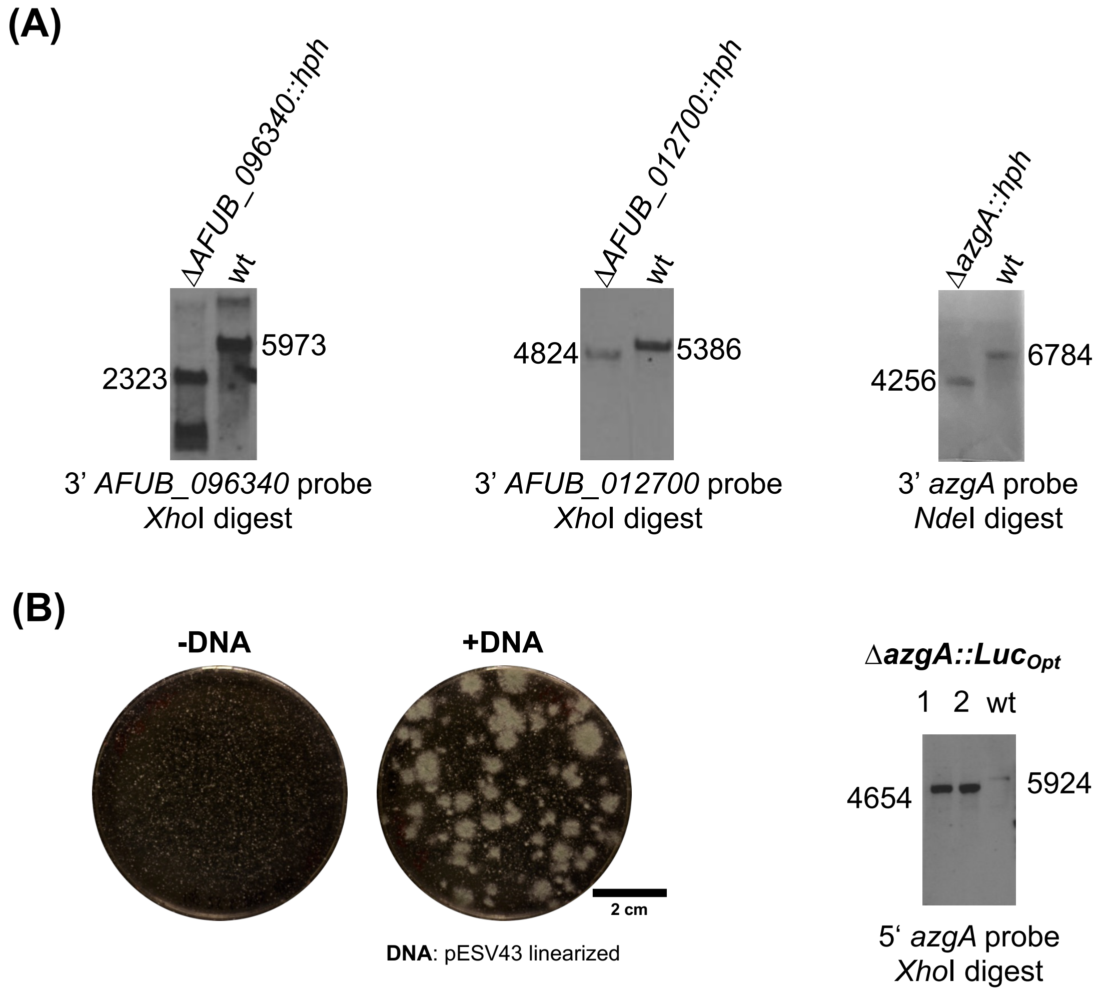

Fig S3 Southern blot analysis of deletion mutants and validation of the single use of *azgA* as counter-selectable marker locus. (A) Putative *A. nidulans azgA* and *furD* orthologs (Δ*AFUB_096340* and Δ*AFUB_012700*) were disrupted using *hph* as selectable marker. For selection hygromycin B was used. (B) *Not*I-linearized pESV43, targeting the *azgA* locus, was transformed into wt. Protoplasts were selected on TOP-AMM pH 5 supplemented with 10 µg/mL 8AG. Replacement of *azgA* was validated by Southern blot analysis in 2 independent transformants. The expected size patterns (bp) for strains are displayed.

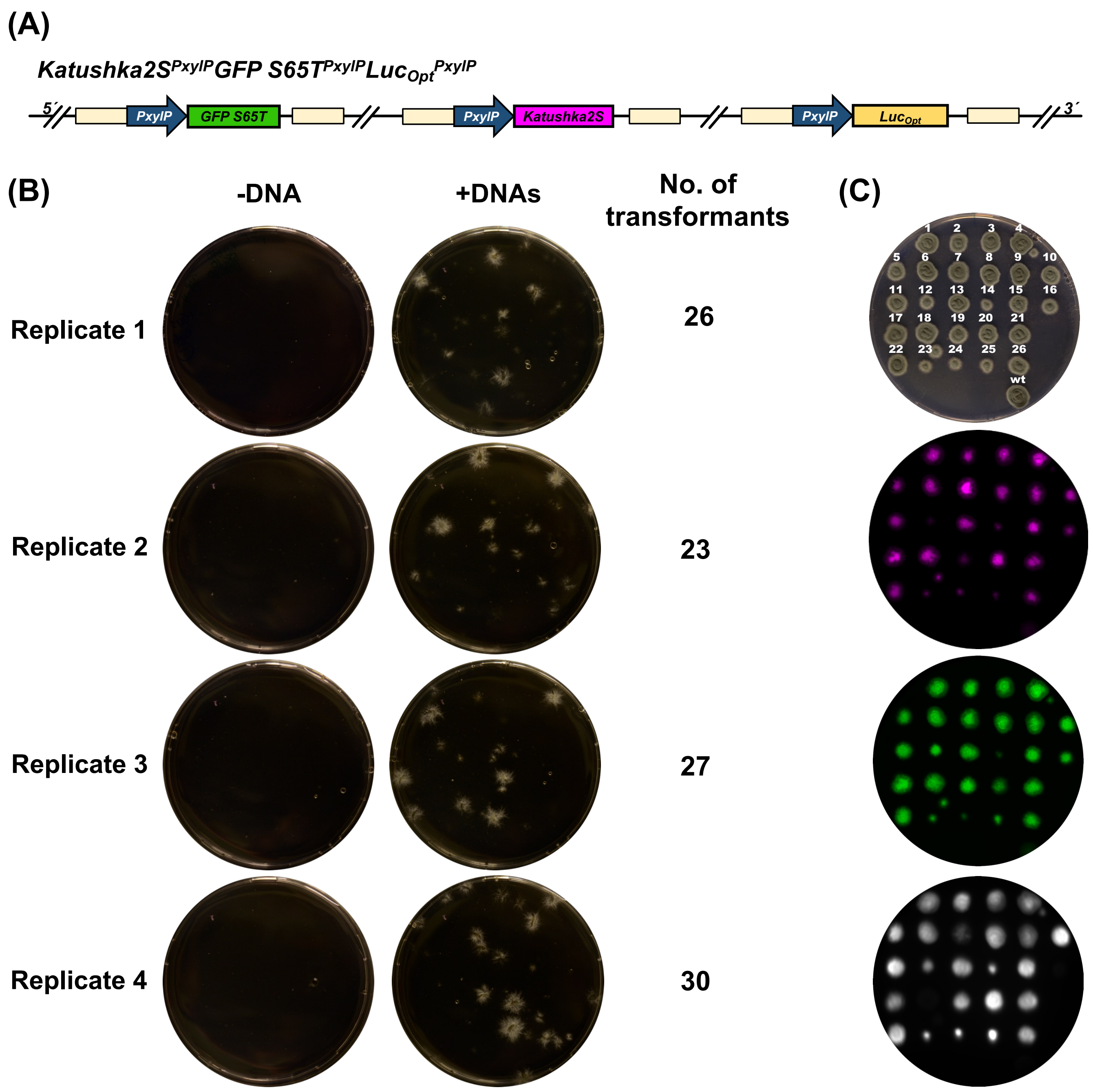

Fig. S4 Simultaneous use of *fcyB*, *cntA* and *azgA* for multigene integration. wt was transformed without (-DNA) with *Not*I-linearized pESV18, pESV34 and pESV43 (+DNA) to generate (A) the multi-reporter strain $Katushka2S^{PxylP}GFP\ S65T^{PxylP}Luc_{Opt}^{PxylP}$. (B) Four independent replica experiments were performed in addition to the first experiment (28 transformants) giving rise to 26, 23, 27 and 30 transformants, respectively. (C) The 26 transformants of replicate 1 underwent a further reporter assay to validate expression of Katushka2S (magenta), GFP S65T (green) and Luc_{Opt} (white). Protoplasts were selected on Top-AMM pH 5 supplemented with 10 µg/mL 5FC, 5FUR and 8AG.

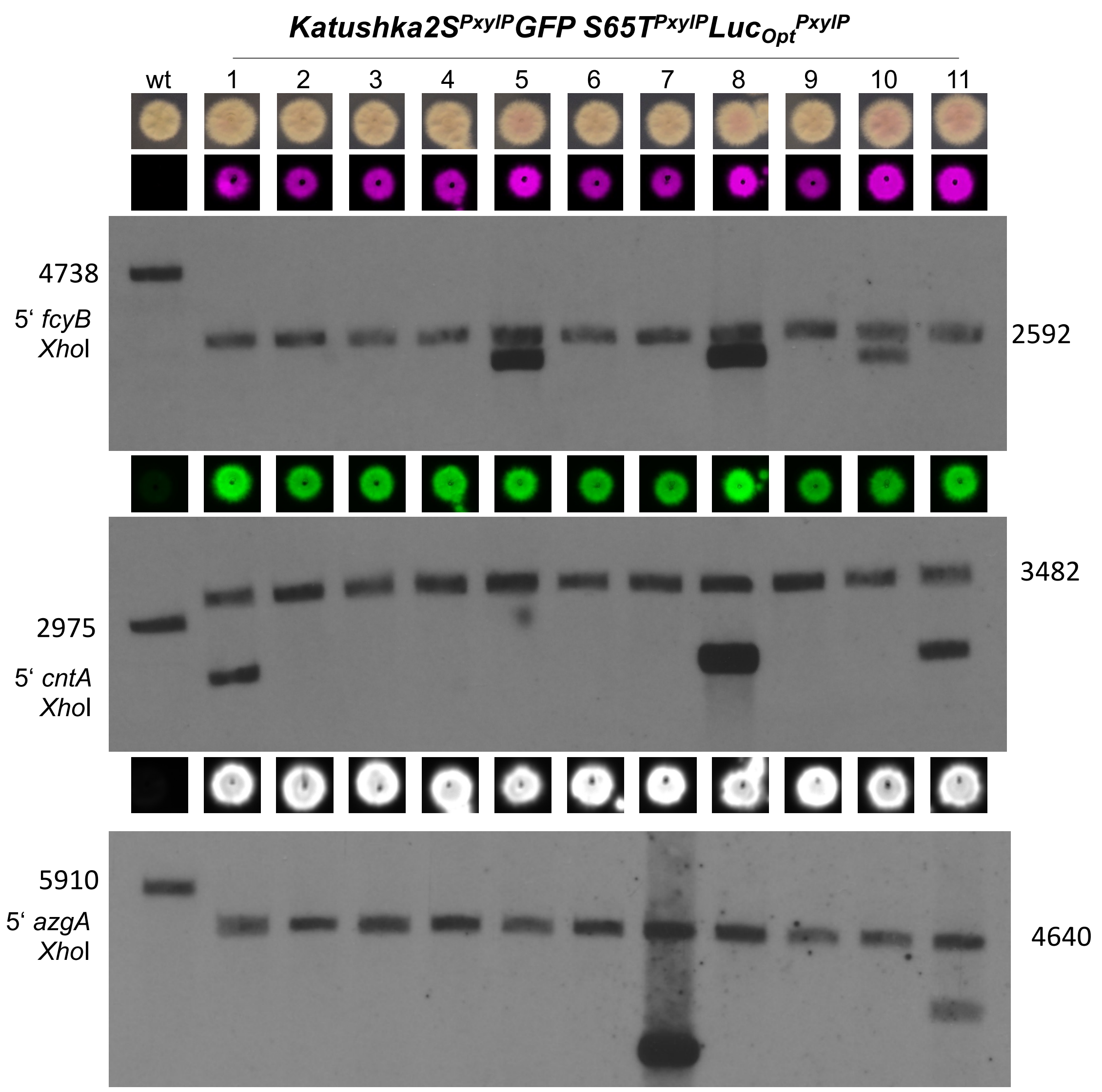

Fig S5 Southern Blot validation of correct, site-directed integration of reporter constructs at the *fcyB*, *cntA* and *azgA* locus. For 11 out of 28 transformants the site-specific integration of constructs was analyzed. The expected size patterns (bp) for wt (left) and successful knock-ins (right) are displayed. Expression of the Katushka2S (magenta), GFP S65T (green) and Luc_{Opt} (white) reporter within colonies is shown.

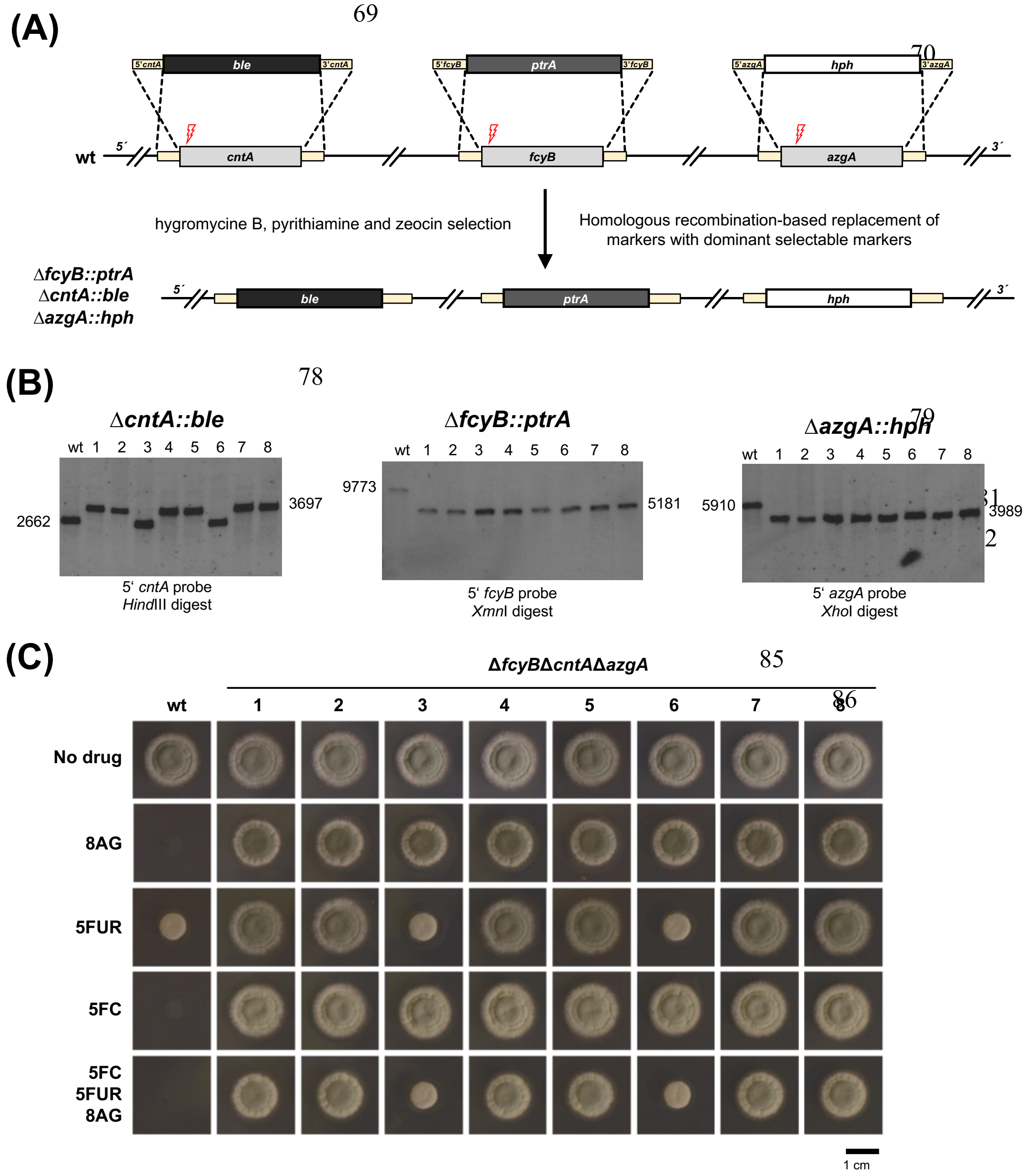

Fig S6 Southern validation of the triple knockout strain Δ*fcyB*Δ*cntA*Δ*azgA* generated with dominant positive selectable markers. A) Graphic illustration of the multiplex strategy followed to generate Δ*fcyB*Δ*cntA*Δ*azgA* using selectable markers *ptrA*, *ble* and *hph*. In 6 out of 8 transformants the expected size patterns (bp) for a triple deletion with the respective resistance cassettes were confirmed (Δ*fcyB::ptrA*Δ*cntA::ble*Δ*azgA::hph*). In 2 (No. 3 and 6) the *cntA* locus was not disrupted. C) All strains displayed 5FC, 5FUR and 8AG resistance patterns that are in agreement with the lack of the individual gene functions of *fcyB*, *cntA* and *azgA*.

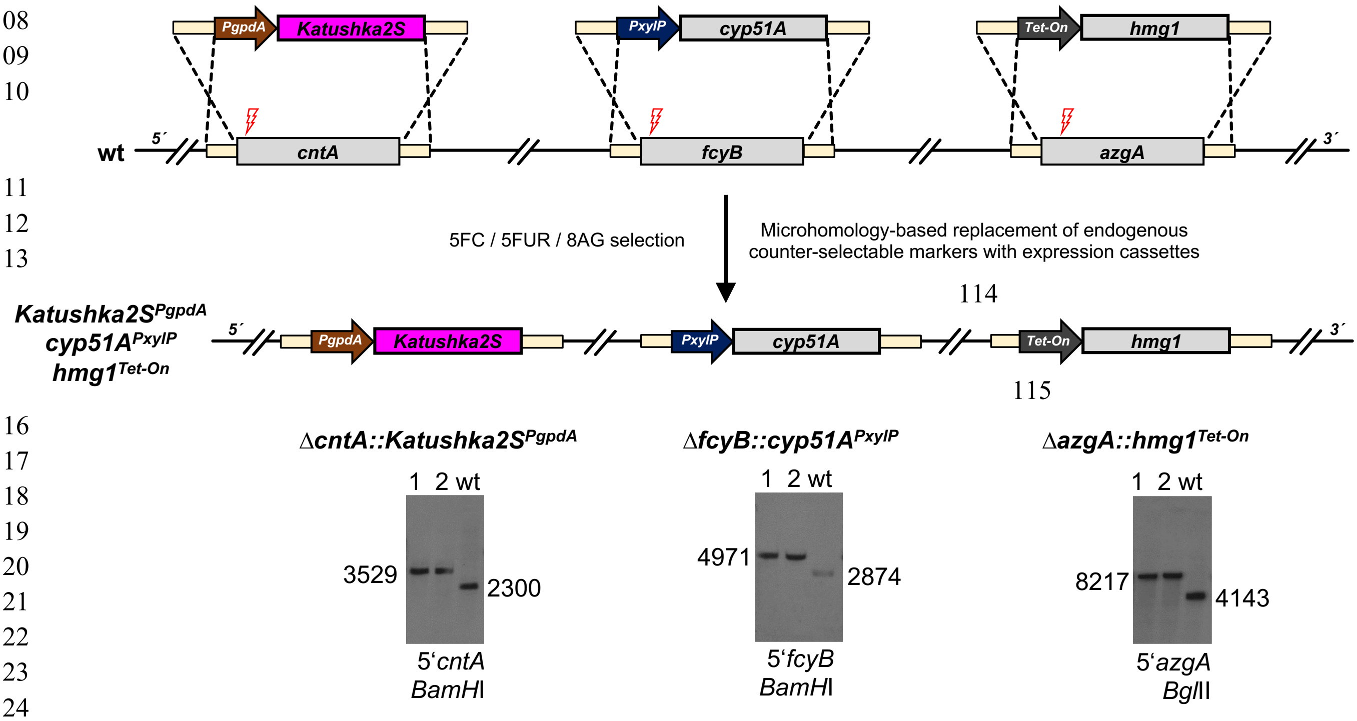

Fig S7 Graphic illustration of the multiplex strategy followed to generate *Katushka2S*^{PgpdA}*cyp51A*^{PxylP}*hmg1*$^{Tet\text{-}On}$. Expression cassettes containing *PgpdA-Katushka2S*, *PxylP-cyp51A* and Tet-On(*PoliC*)-*hmg1* were integrated at target loci *cntA*, *fcyB* and *azgA*, respectively. For this approach, DNA cassettes that served as repair templates were PCR-amplified from plasmids, thereby adding 50 bp overhangs of 5' and 3' regions of the respective target locus for CRISPR/Cas9-assisted microhomology-based recombination. For 2 independent transformants integration at each locus was validated by Southern blot analysis. The expected size patterns (bp) for strains are displayed.

References

1. Fraczek MG, Bromley M, Buied A, Moore CB, Rajendran R, Rautemaa R, Ramage G, Denning DW, Bowyer P: **The cdr1B efflux transporter is associated with non-cyp51a-mediated itraconazole resistance in Aspergillus fumigatus**. *The Journal of antimicrobial chemotherapy* 2013, **68**(7):1486-1496.
2. Gsaller F, Furukawa T, Carr PD, Rash B, Jochl C, Bertuzzi M, Bignell EM, Bromley MJ: **Mechanistic Basis of pH-Dependent 5-Flucytosine Resistance in Aspergillus fumigatus**. *Antimicrob Agents Chemother* 2018, **62**(6).
3. Storer ISR, Sastré-Velásquez LE, Easter T, Mertens B, Dallemulle A, Bottery M, Tank R, Offterdinger M, Bromley MJ, van Rhijn N *et al*: **Shining a light on the impact of antifungals on Aspergillus fumigatus subcellular dynamics through fluorescence imaging**. *Antimicrob Agents Chemother* 2024, **68**(11):e0080324.
